# Thin Layer Immunoassay: An Economical Approach to Diagnose *Helicobacter pylori* Infection in Gastroduodenal Ulcer Disease Patients of Pakistan, a Comparative Analysis

**DOI:** 10.3390/diagnostics13030517

**Published:** 2023-01-31

**Authors:** Faisal Aziz, Shahana Urooj Kazmi

**Affiliations:** 1 The Hormel Institute, University of Minnesota, Austin, MN 55912, USA; 2 Immunology and Infectious Diseases Research Laboratory, Department of Microbiology, University of Karachi, Karachi 75270, Pakistan

**Keywords:** thin-layer immunoassay, *Helicobacter pylori*, gastroduodenal ulcer disease, enzyme-linked immunoassay, *H. pylori* sonicate whole cell antigen, under-developed countries

## Abstract

*Helicobacter pylori* is a causative agent of gastritis, gastroduodenal ulcers and gastric adenocarcinoma. The majority of *H. pylori*-associated patients live in underdeveloped areas, facing the problem of lack of proper diagnostic facility. Hence, a simple and economical assay is required to handle the majority of gastric patients. Serum samples from gastroduodenal ulcers and gastritis patients were screened for *H. pylori* infection by thin layer immunoassay. A polystyrene plate coated with *H. pylori* sonicate whole cell antigen (10 µg/mL). Two-fold diluted patient’s serum was allowed to react at 37 °C, incubated at 60 °C for 1 min over a water bath and the water condensation pattern for the *H. pylori* antibody was recorded. ELISAs were used as reference assays to evaluate the efficacy of the developed thin layer immunoassay (TLI). Gastric patients’ blood samples (62% male and 6% female) tested positive for *H. pylori*, while age-wise, 15–25-year-old males (36%) and 65–75-year-old females (50%) showed the highest number of *H. pylori* infections. TLI showed sensitivity (72–67%), specificity (100%), accuracy (94–69%) and κ value (0.493–0.357) in comparison with wELISA (Surface whole cell ELISA), sELISA (sonicate whole cell ELISA) and kELISA (commercial KIT ELISA). We conclude that thin layer immunoassay is a low cost, fast, simple and clinically reliable method for *H. pylori* diagnosis at initial stages in patients in under-developed countries.

## 1. Introduction

*Helicobacter pylori* is a causative agent of gastritis and ulcer diseases which lead to the development of gastric cancer [1,2,3]. It is a Gram-negative microaerophillic, polysaccharide glycoprotein Lewis antigen (LeY, LeX, LeA, LeB), flagellated and spiral shaped bacilli [4,5,6,7,8]. More than 50% of the world population is colonized by *H. pylori* but few of them suffer from active disease because of various factors such as age, gender, crowding, diet, hyperacidity, smoking habits, unhygienic conditions and poor socio-economic status [9,10,11,12,13,14]. *H. pylori* infection is primarily acquired at a young age and may lead to peptic ulcers or gastric cancer later in life. Non-invasive analytic tools are principally helpful in children as screening checks and for epidemiological studies, but their precision has to be tested against that of invasive tests in patients with symptoms before it can be applied in the general population. Of the available non-invasive tests, serological testing is not precise in young patients and the 13C urea breath test is expensive [15]. The reported prevalence in developing countries is 80% and most infections are acquired during childhood, while in developed countries the reported prevalence rate is 20–50%, which increases later in life [16,17,18]. The developed countries showed an age-related prevalence increase at the ages between 18 and 29 years (10%), 60–69 of years (27%) and 60–69 years (48%), while in developing countries prevalence is not linked to age as 80–90% of the prevalence rate all are observed in adults [19]. Persistent infection of *H. pylori* results in the development of a local chronic inflammatory and systemic antibody response along with secretion and diffusion of large amounts of extracellular products into the mucosa [17,20]. Major humoral and cellular responses against *H. pylori* infection are unable to eliminate it from the host, which results in long-lasting infections and regular systemic immune responses with high antibody levels in patients. Along with other factors, the improper diagnostic facilities are also responsible for increasing the incidence of *H. pylori* infections [11].

There are a number of serological tests for the detection of *H. pylori* antibodies like microagglutination assay, enzyme-linked immunosorbent assay (ELISA) and thin layer immunoassay [21,22]. Multiple factors play a critical role in the selection of the right diagnostic test for the diagnosis of *H. pylori*, including the prevalence of the infection, symptoms, practice, time duration, accessibility and expenditure [22]. ELISA requirements are a mixture of reagents and complicated equipment, but developing countries have difficulty in organizing such costly equipment especially in the rural remote areas of the country [23]. Thin layer immunoassay is a quick and simple assay, which does not require sophisticated equipment and can be carried out by general laboratory equipment and materials at any location [22,24,25]. It is sensitive and based on a simple visualization of antigen–antibody reactions by the naked eye [26,27]. Because of its simplicity, high competence and low cost, it can be used for the screening of a large number of patients’ serum [25]. The aim of this study was to develop and evaluate thin layer immunoassay for rapid and economical diagnosis of *H. pylori* infection in patients with gastroduodenal ulcers in under-developed countries.

## 2. Materials and Methods

### 2.1. Collection, Transport and Processing of Clinical Samples

This is a descriptive study, designed at the Immunology & Infectious Diseases Research Laboratory, Department of Microbiology, University of Karachi in collaboration with the Departments of Medicine and Surgery, Dow University of Health Sciences, Civil Hospital, Karachi, Pakistan, between 2008 to 2014. The inclusion criteria for all cases coming to the endoscopy unit were suffering from gastritis and gastroduodenal ulcer diseases on clinical assessment. The cases included children, younger adults and older persons, aged from 15–75 years old. The cases had already been admitted to the hospital and cases on antibiotic treatment were placed on exclusion criteria. 

Tissue biopsies were obtained from the antral and corpus part of the stomach during gastrointestinal endoscopy (Sydney protocol) along with 214 blood samples. All samples were transported to the Immunology & Infectious Diseases Research Laboratory (IIDRL) for further processing and were frozen at −20 °C until tested. All gastric samples collected from the patients and the research protocols were in accordance with the Karachi University’s Ethical Committee. Gastroduodenal ulcer patients enrolled for gastric biopsy tests were our source of gastric clinical samples, including biopsy tissue, blood and urine samples. 

We collected the sample for protein, RNA extraction and *H. pylori* culture. We transport the sample in transport media and dry ice containers. Later, we processed the biopsy sample for *H. pylori* culture, biochemical tests and PCR reaction. In addition, blood samples were processed for serological assays. In detail, we identified the *H. pylori* status by using different non-invasive (Serology assay) and invasive methods, include bacterial culture, oxidase, catalase, and PCR of Ure, CagA, VacA and 16sRNA from stomach tissue biopsy of gastroduodenal ulcer patient clinical samples. Histopathology results for *H. pylori* provided the data selection for the *H. pylori* negative and positive control. Consent was taken from all participating patients [28].

### 2.2. Preparation of H. pylori Sonicate Whole Cell Antigen

Tissue biopsies were processed and cultured on Colombia agar (CA) plates containing 7% lysed horse blood and antibiotics (Amphotericin B, Trimethoprim, Cefsulodin and Vancomycin). The CA plates were incubated for 4–5 days under microaerophilic conditions at 37 °C and identification was carried out by different conventional biochemical tests (oxidase, catalase and urease test) and molecular methods (PCR of 16sRNA and B-globulin gene). Multiple *H. pylori* strains (e.g., 8 *H. pylori* strain were used based on the *H. pylori* status and pathogenicity level) were inoculated in the broth, incubated at 37 °C for 5 days in a microaerophilic environment. The most virulent strain of *H. pylori* was used to develop a thin layer immunoassay (TLI) assay. The cultured *H. pylori* were centrifuged at 10,000 rpm for 10 min, followed by three washes with 20 mM Tris HCl buffer (pH 7.5) and suspended in the same buffer. The cells were sonicated on ice six times for 30 s with a 60 s interval between each shock. The sonicated samples were centrifuged at 5000 rpm for 10 min to remove the cell debris. Then, the supernatant was treated with 0.5% formalized saline, aliquots were made and kept frozen at −20 °C. Protein concentration was measured by the Bradford assay (Bio-Rad, Hercules, CA, USA), using bovine serum albumin as a standard [29].

### 2.3. Thin Layer Immunoassay

**Figure 1:**
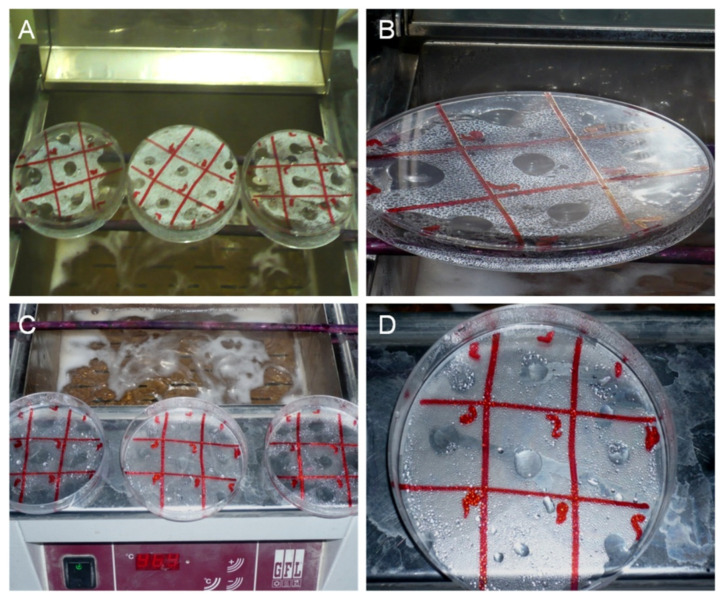
Thin layer immunoassay steps to detect the antibody titer. (**A**) Incubation of polystyrene plate along with different dilutions of patient’s serum over water bath. (**B**) Plate containing droplets after 1 min incubation over bath. (**C**) Water droplets after rinsing with distilled water, blow dried, and again inverted over the water bath at 60 °C. (**D**) Plates showing water droplets regarded as positive results for *H. pylori*.

Polystyrene Petri plates with a hydrophobic surface were rinsed with 70% ethanol and air dried (dry with a jet of air). A BSA solution (0.05 M NaHOC3 (pH 9.6) as diluent) (20 mL) containing 10 µg/mL of *H. pylori* sonicate whole cell antigen was coated on plate and incubated overnight at 4 °C. The antigen solution was poured off and the plate was washed thoroughly and gently three times with 150 mM NaCl solution and with distilled water and finally air dried. A grid was drawn on the backside of the plate to provide multiple squares. Two-fold serial dilutions of each of the 214 human gastric patients’ antiserum (1:2 and 1:16384) was prepared in NaC1 (0.15 M); 5 µL of the antiserum dilution was spotted on the respected grid of the plate using a capillary tube, followed with incubation in a humid chamber at 37 °C for 1 h (Supplementary Table S1). The plate with serum drops was inverted over a water bath at 60 °C for one minute. Later, the plate was rinsed thoroughly with distilled water, blow dried and again inverted over the water bath at 60 °C for 1 h. Small drops or condensation patterns were observed on the place where the serum was placed which were regarded as antigen/antibody reactions (Figure 1). The dilutions showing water droplets larger than droplets appearing in the background were recorded as positive results. Normal control serum was used as negative controls and infected patient serum was used as positive controls. In addition, the concentration and conditions for incubation of the reagents were optimized. In order to determine the optimal conditions of TLI, different concentrations of antigen and buffer solutions (PBS, 0.05% Tween in PBS, distilled water, 0.85% NaCl solution, and 0.1 M carbonate buffer) were tested. Polystyrene plastic plates were coated with different sets of positive and negative sera (Supplementary Table S2). In addition, other factors of the assays were also optimized, including time of incubation, buffer compositions and concentration, temperature for incubation of the antigen, antiserum and other reagents to determine the best optimized parameters for TLI.

### 2.4. Confirmation of Antigen/Antibody Reaction

For further confirmation of the antigen/antibody reaction in the TLI assay, we added 10 mL of secondary antibody horseradish peroxidase conjugated goat anti-mouse IgG (H + L) [Bio-Rad Holland] diluted 1:40 in PBS-T and incubated for 1 h at 37 °C. Later, 5 µL of TMB one solution (Tetramethylbenzidine) substrate (G7431, Promega, USA) was added exactly on the antigen spot and incubated for 30 min at room temperature. The reaction turned into colored dots which were detected by the naked eye. The presence of colored reaction zones was the criterion for positivity of the test. The intensity of reaction was arbitrarily classified as weak, medium and strong. Multiple controls were used as positive and negative controls which were validated by other non-invasive and invasive assays.

### 2.5. Anti-H. pylori IgG and IgM Titer by Commercial ELISA Kit (kELISA)

Serological assessment of *H. pylori* infection was measured by a commercial ELISA kit (Equipar VA, Italy). Commercial ELISA kits were used to analyze *H. pylori* titer values according to the manufacturer’s instructions. A total of 100 μL (1:101 diluted) of patient serum was added into consecutive wells along with negative and positive controls and incubated at 37 °C for 1 h. After extensive washing, HRP-conjugated anti-human IgM and IgG antibodies (1:20) were added and incubated for 1 h at 37 °C. Later, 100 μL of chromogen tetramethylbenzidine (TMB) and hydrogen peroxide (H_2_O_2_) substrates (1:1) was added and kept for 20 min at room temperature in a dark place. The reaction was stopped by adding a stop solution (1:100 μL), and the color intensity was measured using an ELISA reader (STAT FAX-2100). The data were statistically analyzed using the commercial kit’s cutoff value [28].

### 2.6. Statistical Analysis 

All statistical analyses were performed using Graphpad Prism 5.0 software (San Diego, CA, USA), with differences between groups considered significant with a *p* value < 0.05. Data are presented as mean values ± S.E.M. The serological parameters (sensitivity, specificity, accuracy and predictive value for a positive and for a negative result, as well as the kappa coefficient) were optimized and analyzed for the thin layer immunoassay. These data were determined in order to compare with other serological assays by Dohoo 2004. The comparative analysis between the thin layer immunoassay and previously reported serological assays such as in-house ELISA based on *H. pylori* surface whole cell antigen (wELISA) [28], in-house ELISA based on *H. pylori* sonicate whole cell antigen (sELISA) [29] and an ELISA commercial kit (kELISA) [28] were assessed by the κ statistic. 

### 2.7. Ethical Clearance

This study was approved by the Karachi University’s Ethical Committee in Karachi, Pakistan.

## 3. Results

This study was designed to develop a thin layer immunoassay for the diagnosis of *H. pylori* infection in gastritis and gastroduodenal patients of Karachi, Pakistan. A total of 214 blood samples were collected from patients reporting at the Civil Hospital Karachi with symptoms of gastritis and gastroduodenal problems. Blood collected from male or female patients (35% and 65%, respectively) was processed in the IIDRL (Immunology & Infectious Diseases Research Laboratory), Department of Microbiology, Karachi.

### 3.1. Analysis of H. pylori Titer by Thin Layer Immunoassay

**Figure 2:**
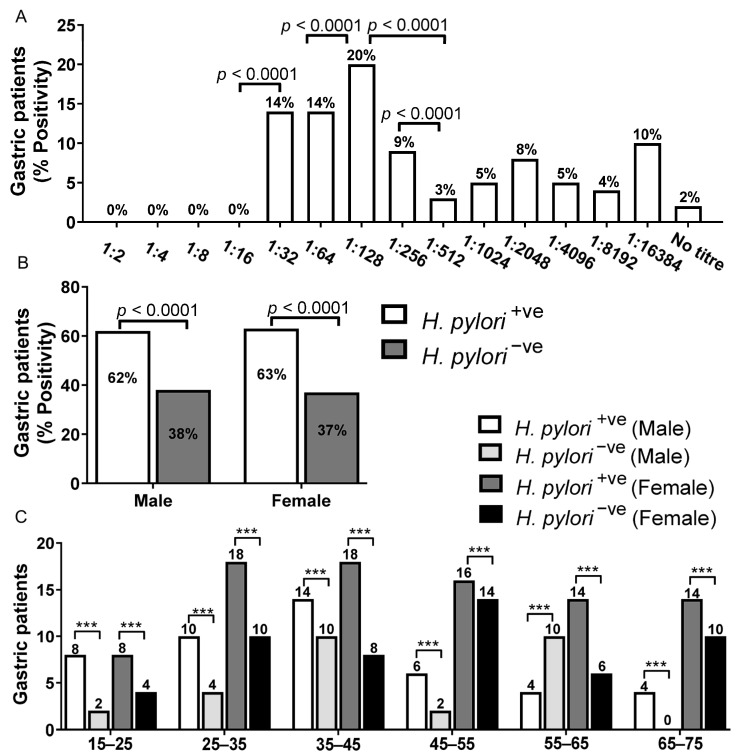
(**A**) Comparison of anti-*H. pylori* antibody titers between different positive percentages of gastroduodenal ulcer and gastritis patients by thin layer immunoassay. (**B**) Bar diagram showing high number of male positive results compared to female by TLI method. (**C**) Age wise distribution (15–75 years) of *H. pylori* infected gastric patients by thin layer immunoassay in reference to sex. Three asterisks (***) indicate *p* value smaller than 0.001 (*p*,0.001).

A total of 214 human sera samples were screened for anti-*H. pylori* antibodies by thin layer immunoassay (Figure 1). The majority of the sera from gastric patients showed a low percentage anti-*H. pylori* titer. We found that 20% of patients have 1:128 anti-*H. pylori* titer, while 14% showed 1:32 and 1:64 titer value. The highest dilution of anti-*H. pylori* (1:16384) was found in 10% of gastric patients. However, titers of 1:16–1:2 showed 0% positive patients, while 2% samples showed no titer and 3% and 4% showed high titers (1:512 and 1:8192) (Figure 2A). These data showed that 1:512 could be clear cut-off value to differentiate the *H. pylori* status in the gastric patient samples (Supplementary Table S1).

### 3.2. Age and Sex Distribution of Gastritis and Gastroduodenal Ulcer Patients by Thin Layer Immunoassay

In this study, 214 patients were enrolled between the age of 15 and 75 years (65% females and 35% males). Overall seropositivity of *H. pylori* infection was found to be 63% by TLI as compared to 87% by wELISA [28] and 92% by sELISA [29]. Female (63%) and male (62%) patients were found to be *H. pylori* seropositive with sex-wise distribution (Figure 2B). However, age-wise distribution showed slight differences in the risk of seropositive *H. pylori* in gastric patients. We found a high risk of infection between the younger age groups of 15–25 years (73%) and 25–35 years (67%) as compared to the middle ages of 45–55 years (58%), and 55–65 years (53%) have a low risk of infection. Male patients (15–25 years) were shown to have a high risk of infection (36%), while older patients (55–65 years) showed low (12%) prevalence of *H. pylori* infections. In contrast, the highest risk of infection in the female population was found between the ages of 65–75 years (50%). Female patients (15–65 years) showed a moderate risk of infection (36–43%) (Figure 2C).

### 3.3. Analysis of Anti-H. pylori IgM and IgG Titer by Commercial Kit ELISA (kELISA)

**Figure 3:**
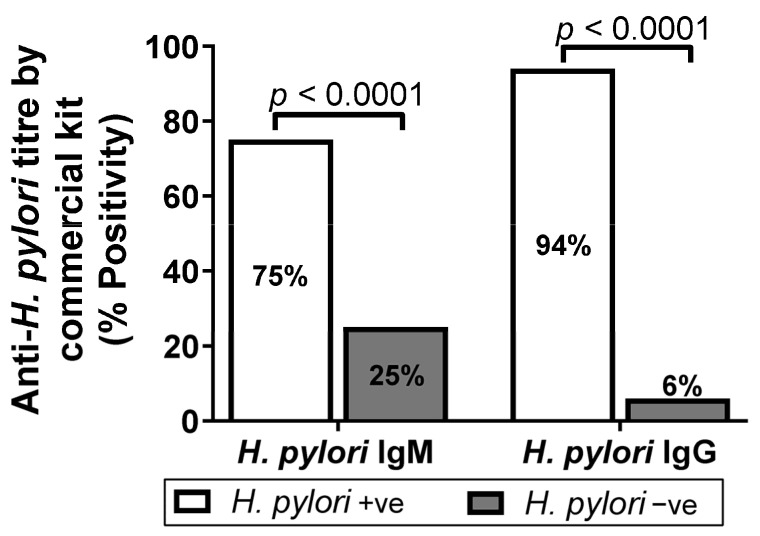
Graph showing comparison of *H. pylori* IgG and IgM titer between different *H. pylori* positive percentages of gastroduodenal ulcer and gastritis patients using commercial kit ELISA.

In order to determine the reliability of wELISA and sELISA, we determined anti-*H. pylori* IgM and IgG titer using a commercial ELISA kit. We found 75% (0.921 ± 0.633) and 94% (2.144 ± 0.997) of gastritis patient serum samples were seropositive for IgM and IgG, respectively (Supplementary Tables S3 and S4 and Figure 3) on the basis of respected cut0ff value = Mean of negative control + 0.250.

### 3.4. Age- and Sex-Wise Distribution of Gastric Patients IgM and IgG Titer by Commercial Kit ELISA (k_M_ELISA and k_G_ELISA)

**Figure 4:**
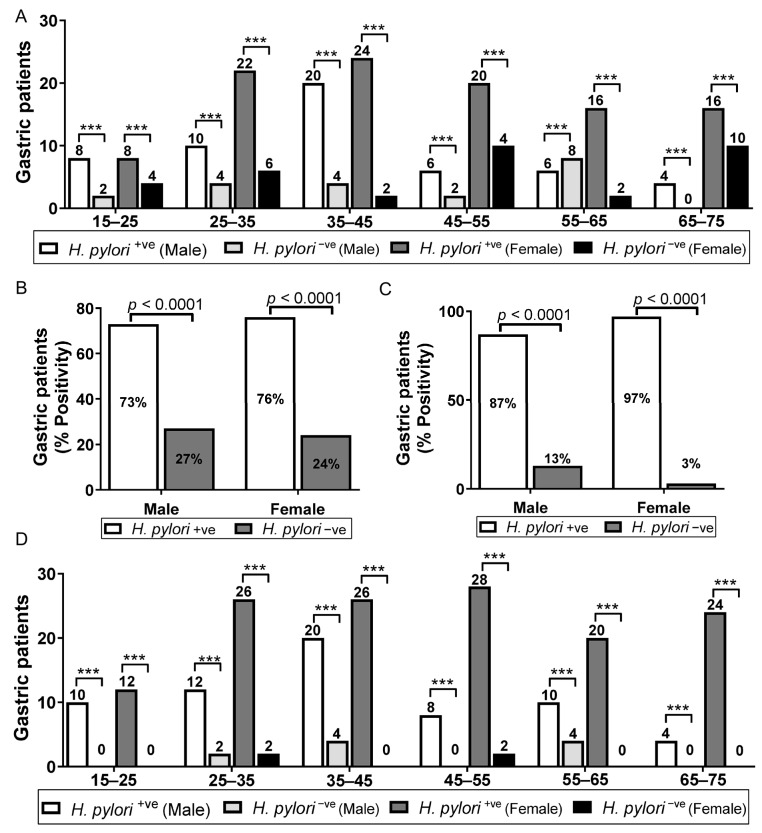
(**A**) Age-wise distribution of *H. pylori* infections in gastroduodenal ulcer and gastritis patients by IgM commercial ELISA kit (k_M_ELISA). (**B**) Sex-wise distribution of *H. pylori* infections in gastroduodenal ulcer and gastritis patients by IgM commercial ELISA kit (k_M_ELISA). (**C**) Sex-wise distribution of *H. pylori* infections in gastroduodenal ulcer patients and gastritis by IgG commercial ELISA kit (k_G_ELISA). (**D**) Age-wise distribution of *H. pylori* infections in gastroduodenal ulcer patients and gastritis by IgG commercial ELISA kit (k_G_ELISA). Three asterisks (***) indicate *p* value smaller than 0.001 (*p*,0.001).

In this study, 214 patients were enrolled between the age of 15 and 75 years (65% of females and 35% of males); 75% of the patients were found to be *H. pylori* seropositive (Figure 3), while sex-wise distribution was female (76%) and male (73%) (Figure 4A). We observed a high risk of *H. pylori* seropositivity in all patients with little differences. Age-wise distribution showed a high risk of infection between the ages of 35 and 45 years (88%) as compared to 65–75 years (66.66%) who had a low risk of infections. Male patients (15–25 years) showed high seropositivity (36.36%) compared to 65–75-year-old patients who showed low seropositivity (13.33%). The highest risk of infection (53.33%) in the female population was observed between the ages of 65 and 75 years, while 15–25 years patients showed low seropositivity (36.36%) (Figure 4B). However, 94% of the patients were found to be *H. pylori* seropositive by the commercial IgG ELISA kit (Figure 3), with the sex-wise distribution as female (97%) and male (87%) (Figure 4C). We observed a high risk of *H. pylori* seropositivity between the ages of 15–25 and 65–75 years (100%) as compared to ages of 55–65 years (88.2%) which had a low risk of infection. Male patients (15–25 years) were shown to have the highest seropositivity (45.45%) while an apparent decrease in the risk of infection (14.28%) was observed in 65–75-year-old patients. The highest risk of infection in the female population was found between the ages of 65 and 75 (85.71%) (Figure 4D).

### 3.5. Evaluation of TLI with Other Serological Assays

In order to evaluate the authenticity of TLI, we analyzed its sensitivity, specificity and accuracy and compared it with previously reported assays from the IIDRL (Immunology & Infectious Diseases Research Laboratory), such as an ELISA based on *H. pylori* surface whole cell antigen (wELISA) [28], ELISA based on *H. pylori* sonicate whole cell antigen (sELISA) [29] and a commercial ELISA kit (kELISA) [28]. We found moderate agreement of TLI with wELISA (κ value = 0.493) and fair agreement with sELISA (κ value = 0.396) and kELISA (κ value = 0.357). TLI had a sensitivity of 72% in comparison with wELISA, 68% with sELISA and 67% with kELISA. Moreover, we found a 76% accuracy of TLI in comparison with wELISA, 71% with sELISA and 69% with kELISA. TLI did not produce false positive values while negative predictive values were found in the range of 18–35%. Based on the accuracy, we can say that the diagnostic value of TLI was fair (76–71% = fair test) (Table 1). 

## 4. Discussion

*Helicobacter pylori* is a non-invasive bacterium which stimulates the immune response by releasing different immunogenic proteins and lipopolysaccharides [30]. ELISA performance is mainly based on the nature of the antigen and *H. pylori* strain [31]. *H. pylori* has different types of antigen candidates, such as urease, catalase, CagA, VacA, BabA, HspA, the FliD protein and multivalent epitopes [32]. There are several protocols for *H. pylori* antigen preparation, including formaldehyde- or heat-treated whole bacteria, sonic extract and acid glycine extract. Whole cell lysate is the best choice for antigen preparation as compared to a purified antigen which is unable to be recognized by various types of antibodies present in the population. Different populations may harbor different types of *H. pylori* antigens [33]. Local *H. pylori* strain antigens are useful with enough influence on the diagnostic properties of serological assay [28].

Serological analysis of *H. pylori* infection is a non-invasive and less expensive method [31]. There are many serological assays for *H. pylori* detection which differs on the basis of their sensitivity [11,22]. These methods have high sensitivities and specificities, yet the expense, time and expertise have led to a search for an economic, quick and simple serological assay, which is applicable and reliable for under-developed countries. Thin layer immunoassay is attractive immunoassay in comparison with ELISA because of its quick result, low cost and simplicity. There is no requirement for sophisticated equipment and standardized reagents, such as enzyme-conjugated antibody and substrate and the results can be examined by the naked eye [23]. Hence, it is appropriate to use to diagnose *H. pylori* infection in gastritis and gastroduodenal ulcer patients from remote areas, where hospital and laboratory facilities are not well developed. In order to study the seroprevalence of *H. pylori* infection in the population from a rural part of Pakistan, we investigated the immune response in gastric patients by developing a TLI from the local strain of *H. pylori*. The overall age-wise prevalence rate of *H. pylori* infection was highest among the young age group of 15–25 years (73%) with 25–35 years (67%) showing the highest rate of *H. pylori* seropositivity. Edity el. al., 2021 showed the highest rate of infection between 19- to 35-year-old patients [34]. However, middle-age and older people showed a low incidence of *H pylori* infection. These results are consistent with the reported data of high rates of *H. pylori* infection in younger gastric patients [35,36,37]. 

Antibody titer represents meaningful data to verify the disease status and is useful for antibody screening. *H. pylori* antibody indicates gastric colonization which may be used as a tool to screen large numbers of gastric patients. In this study, we found an overall moderate prevalence rate (63%) while the sex-wise distribution showed little variation between females (63%) and males (62%). According to Amjad et al., Pakistan’s population has an *H. pylori* prevalence of approximately 80.5% between 1993 and 1997 [11]. In addition, TLI showed the highest titer (1:1,6384) in 22 (10%) patients while 42 (20%) showed a 1:128 titer of *H. pylori*. A low number of patients (6, 3%) showed 1:512 titers.

Commercial ELISA kits showed high sensitivity because they use purified antigens at the most suitable concentration. In this study, we found the overall anti-*H. pylori* IgG titer to be 94% while anti-*H. pylori* IgM titer was found to be 75%. A similarly high rate was previously reported by other researchers. Crabtree et al. reported the sensitivity of *H. pylori* commercial ELISA kits to be between 100–94% with a specificity of 85–67% [38]. According to Alem et al., an IgM response was raised after 18 days of *H. pylori* ingestion, but IgG and IgA sero-conversion after 60 days led to decreased IgM titer [39]. Hence, IgG and IgA titers may indicate the post-exposure or active infection and suggests testing the *H. pylori* infection patients’ IgG and IgA titers [39]. Commercial ELISA kits are too expensive to use to diagnose a large population of patients with *H. pylori* infection in developing countries and the heterogenicity of *H. pylori* strains also limits the use of commercial ELISA kits. Moreover, the diagnosis accuracy of commercial ELISA kits in Asian countries is low compared to the Western countries because of the heterogenicity of *H. pylori* strains [40], which indicated that in-house ELISA based on local *H. pylori* isolates will result in higher sensitivity and accuracy. Hence, the best alternative is to develop in-house ELISAs using local *H. pylori* strains, which reduces the heterogenicity issue of *H. pylori* strains and the diagnostic cost in developing countries.

TLI based on sonicated whole cell antigen were developed to confirm the *H. pylori* infection with a high degree of specificity and sensitivity. We compared thin layer immunoassay with other reported immunoassays that were applied in our laboratory to demonstrate the variability in sensitivity, specificity and other evaluation parameters. Sensitivity can be affected by many factors, such as concentration of reactants, the capacity of the solid phase, the assay speed and incubation temperature. The elevated and long incubation temperature resulted in the improvement of sensitivity, but the dissociation rate and intra-assay variation can also be high. In the comparison of TLI with wELISA [28], sELISA [29] and kELISA [28], we found a low sensitivity of 72%, 68% and 67%, respectively. However, the accuracy of TLI was in the range of 69–76% in comparison with wELISA, sELISA and kELISA (Table 1). There was a moderate association between TLI and wELISA (κ value = 0.493); however, there was only a fair association with sELISA (κ value = 0.396) and kELISA (κ value = 0.357). Gomez et al., reported high sensitivity and kappa values of thin layer immunoassay compared to ELISA, indicating that thin layer immunoassay can identify more positive samples than ELISA [22]. Ismail et al. reported the comparable sensitivity with low specificity of thin layer immunoassay in comparison with ELISA [24]. According to Nilsson et al., thin layer immunoassay seemed to be less sensitive because of its simplicity and high capacity [25]. False positive test results may be due to the presence of cross-reacting bacterial antigens [41] that can be reduced by an adsorption assay with species that are closely related to *H. pylori* [33]. In our study, we did not find any false positive results; hence, no absorption step was considered necessary. The sensitivity, specificity and accuracy indicated TLI is a fair test to detect *H. pylori* antibody titers in patients with gastritis and ulcer diseases. Commercial ELISA kits are so expensive and have no surety of high specificity and sensitivity because of *H. pylori* strain variations. *H. pylori* heterogenicity and cost of commercial ELISA kits limited their application, while in-house serological assays have no such problems [28].

The simplicity, low cost and rapid results of TLI makes its selection as the best assay for early diagnosis of *H. pylori* in clinical laboratories, field surveys and rural areas. Furthermore, a large number of samples can be diagnosed because of its rapid screening ability [42]. TLI has the advantage of giving qualitative as well as quantitative information concerning antibody content at a low cost. However, TLI has the disadvantage of requiring a high amount of antigen, no use of a second antibody reaction and no proper indicator. Furthermore, TLI showed less sensitivity and specificity compared to ELISA. TLI could be suitable for the diagnosis of *H. pylori* infection at the initial stage of diagnosis. The simplicity and sensitivity of the TLI assay make it acceptable for the diagnosis of a wide range of other infections, including Trichinellosis [22,43], echinococcosis [44], S. huematobiurn and S. mansoni [24] and Trypanosoma cruzi [45]. TLI showed encouraging results as a preliminary diagnosis test for infection. Hence, TLI could be widely used for different types of infection with little modification. The TLI method provides an easy, economical and simple way of diagnosing infections.

The area recorded is directly related to the logarithmic expression of the antibody concentration [25]. The visualization procedure of TLI is one of its advantages as an antigen/antibody reaction may be detected by the naked eye, based on the principle of vapor condensation indicating an area of antigen/antibody reaction due to containing high protein levels and is more hydrophilic than the surrounding reaction [42,46]. The property of many antigens to become adsorbed firmly onto a hydrophobic polystyrene surface while retaining their serological reactivity is taken advantage of here. On a surface with adsorbed antigen, the corresponding immune serum is applied spot-wise. The antigen–antibody reaction areas on the surface are characterized by a distinct hydrophilic condensation pattern when exposed to water vapor. The results obtained by the described immunoassay technique can be reproduced with great accuracy. The method is well suitable for quantitative determination of a wide range of antigens as well as their corresponding antibodies [26]. The result of TLI can be saved by preserving the polystyrene plate and this storage did not cause any false positive or false negative results, even though there was an apparent growth of microorganisms in the samples [46]. However, TLI has the disadvantage of requiring a higher concentration of antigens and cannot be applied for high antibody titer detection [24].

## 5. Conclusions

An ideal serodiagnostic test, especially for under-developed countries, should be low cost, rapid and easy to perform, in addition to being sensitive and specific. TLI fulfills these requirements and is suitable to be used in field studies to get medical attention at the right time, as required in outbreaks of infection. We conclude that TLI is a reliable, low cost, simple and clinically useful assay to diagnose *H. pylori* infection in gastric patients in under-developed countries.

## Figures and Tables

**Table 1:** Table showing comparative analysis of thin layer immunoassay with other comparable serological assays [28,29].

Standard Assays	Sensitivity %	Specificity %	Accuracy %	PPV %	NPV %	FPV	FNV	OA	EA	AABC	PABC	KAPPA
	*H. pylori* Thin Layer Immunoassay											
wELISA	72	100	76	100	35	0.0	0.28	0.757	0.52	0.237	0.480	0.493
sELISA	68	100	71	100	22.5	0.0	0.316	0.710	0.52	0.190	0.480	0.396
kELISA	67	100	69	100	18	0.0	0.33	0.691	0.52	0.17	0.480	0.357

## Data Availability

The data that support the findings of this study are available on reasonable request from the corresponding author.

## Supplementary Materials

The following supporting information can be downloaded at: https://www.mdpi.com/article/10.3390/diagnostics13030517/s1, Table S1: *Helicobacter pylori* antibody titre in patients suffering from gastroduodenal ulcer and gastritis is determined by thin layer immunoassay method; Table S2: Different type of negative and positive used to develop thin layer immunoassay; Table S3: Anti-*H. pylori* IgM antibody titer values in gastroduodenal ulcer and gastritis patients by IgM commercial kit ELISA (k_M_ELISA); Table S4: Anti-*H. pylori* IgG antibody titer values in gastroduodenal ulcer and gastritis patients by IgG commercial kit ELISA (k_G_ELISA).

## Abbreviations

ELISA	Enzyme-linked immunosorbent assay
*H. pylori*	*Helicobacter pylori*
kELISA	ELISA commercial kit
OD	Optical density
wELISA	In-house ELISA based on *H. pylori* surface whole cell antigen
sELISA	In-house ELISA based on *H. pylori* sonicate whole cell antigen
