# Supplementary material for: Thin Layer Immunoassay: An Economical Approach to Diagnose Helicobacter pylori Infection in Gastroduodenal Ulcer Disease Patients of Pakistan, a Comparative Analysis
Source: Diagnostics (Basel). 2023 Jan 31;13(3):517. doi: 10.3390/diagnostics13030517 (PMC9914287; doi:10.3390/diagnostics13030517)
Supplement: Supplementary file 1 [file diagnostics-13-00517-s001.zip › diagnostics-2165948-supplementary.pdf]

Supplementary Table S1: ***Helicobacter pylori*** **antibody titre in patients suffering from gastroduodenal ulcer and gastritis is determined by thin layer immunoassay method**

S.No	SAMPLE CODE	C1	C2	1:2	1:4	1:8	1:16	1:32	1:64	1:128	1:256	1:512	1:1024	1:2048	1:4096	1:8192	1:16384
1.	A1	-	+	-	-	-	-	-	-	-	-	-	-	-	-	-	-
2.	A2	-	+	-	-	-	-	-	-	-	-	-	-	-	-	-	-
3.	2	-	+	+	+	+	+	+	+	+	+	+	+	+	-	-	-
4.	3	-	+	+	+	+	+	+	+	+	+	+	+	+	+	+	-
5.	5	-	+	+	+	+	+	+	+	+	+	+	+	+	-	-	-
6.	6	-	+	+	+	+	+	+	+	+	+	-	-	-	-	-	-
7.	7	-	+	+	+	+	+	+	+	+	+	-	-	-	-	-	-
8.	11	-	+	+	+	+	+	+	+	+	+	+	+	+	-	-	-
9.	12	-	+	+	+	+	+	+	+	+	-	-	-	-	-	-	-
10.	13	-	+	+	+	+	+	+	+	+	+	+	+	+	+	+	-
11.	14	-	+	+	+	+	+	+	+	+	-	-	-	-	-	-	-
12.	15	-	+	+	+	+	+	+	+	+	+	+	+	+	+	-	-
13.	16	-	+	+	+	+	+	+	+	+	-	-	-	-	-	-	-
14.	17	-	+	+	+	+	+	+	+	+	-	-	-	-	-	-	-
15.	18	-	+	+	+	+	+	+	+	+	+	+	+	+	+	+	-
16.	19	-	+	+	+	+	+	+	+	-	-	-	-	-	-	-	-
17.	21	-	+	+	+	+	+	+	+	-	-	-	-	-	-	-	-
18.	22	-	+	+	+	+	+	+	+	-	-	-	-	-	-	-	-
19.	23	-	+	+	+	+	+	+	-	-	-	-	-	-	-	-	-
20.	24	-	+	+	+	+	+	+	+	+	+	+	+	+	+	+	+
21.	25	-	+	+	+	+	+	+	+	+	+	-	-	-	-	-	-
22.	26	-	+	+	+	+	+	+	+	+	-	-	-	-	-	-	-
23.	27	-	+	+	+	+	+	+	+	+	+	+	+	+	+	-	-
24.	28	-	+	+	+	+	+	+	+	+	+	-	-	-	-	-	-
25.	29	-	+	+	+	+	+	+	-	-	-	-	-	-	-	-	-
26.	34	-	+	+	+	+	+	+	-	-	-	-	-	-	-	-	-
27.	35	-	+	+	+	+	+	+	+	+	-	-	-	-	-	-	-
28.	36	-	+	+	+	+	+	+	+	-	-	-	-	-	-	-	-
29.	37	-	+	+	+	+	+	+	+	-	-	-	-	-	-	-	-
30.	38	-	+	+	+	+	+	+	+	+	+	-	-	-	-	-	-
31.	39	-	+	+	+	+	+	+	+	-	-	-	-	-	-	-	-

32.	40	-	+	+	+	+	+	+	+	+	+	+	+	+	+	-	-
33.	41	-	+	+	+	+	+	+	+	+	+	-	-	-	-	-	-
34.	43	-	+	+	+	+	+	+	+	+	+	-	-	-	-	-	-
35.	44	-	+	+	+	+	+	+	+	+	+	+	+	+	-	-	-
36.	48	-	+	+	+	+	+	+	-	-	-	-	-	-	-	-	-
37.	49	-	+	+	+	+	+	+	-	-	-	-	-	-	-	-	-
38.	50	-	+	+	+	+	+	+	+	+	+	-	-	-	-	-	-
39.	51	-	+	+	+	+	+	+	+	-	-	-	-	-	-	-	-
40.	52	-	+	+	+	+	+	+	+	+	-	-	-	-	-	-	-
41.	53	-	+	+	+	+	+	+	+	-	-	-	-	-	-	-	-
42.	54	-	+	+	+	+	+	+	+	+	-	-	-	-	-	-	-
43.	55	-	+	+	+	+	+	+	+	+	+	+	+	-	-	-	-
44.	56	-	+	+	+	+	+	+	+	+	+	+	+	+	-	-	-
45.	59	-	+	+	+	+	+	+	+	-	-	-	-	-	-	-	-
46.	60	-	+	+	+	+	+	+	+	-	-	-	-	-	-	-	-
47.	61	-	+	+	+	+	+	+	-	-	-	-	-	-	-	-	-
48.	67	-	+	+	+	+	+	+	+	+	-	-	-	-	-	-	-
49.	69	-	+	+	+	+	+	+	+	+	+	+	+	-	-	-	-
50.	70	-	+	+	+	+	+	+	+	+	+	+	+	-	-	-	-
51.	71	-	+	+	+	+	+	+	-	-	-	-	-	-	-	-	-
52.	74	-	+	+	+	+	+	+	+	+	+	+	+	+	+	+	+
53.	75	-	+	+	+	+	+	+	-	-	-	-	-	-	-	-	-
54.	77	-	+	+	+	+	+	+	+	+	+	+	+	+	+	-	-
55.	78	-	+	+	+	+	+	+	+	+	+	+	+	+	+	+	+
56.	80	-	+	+	+	+	+	+	-	-	-	-	-	-	-	-	-
57.	86	-	+	+	+	+	+	+	-	-	-	-	-	-	-	-	-
58.	87	-	+	+	+	+	+	+	+	-	-	-	-	-	-	-	-
59.	88	-	+	+	+	+	+	+	+	+	+	+	+	+	+	+	+
60.	89	-	+	+	+	+	+	+	+	+	+	+	+	+	+	+	-
61.	92	-	+	+	+	+	+	+	+	+	-	-	-	-	-	-	-
62.	95	-	+	+	+	+	+	+	+	+	-	-	-	-	-	-	-
63.	97	-	+	+	+	+	+	+	+	+	-	-	-	-	-	-	-
64.	99	-	+	+	+	+	+	+	+	-	-	-	-	-	-	-	-
65.	10s	-	+	+	+	+	+	+	+	-	-	-	-	-	-	-	-

66.	11s	-	+	+	+	+	+	+	+	+	-	-	-	-	-	-	-
67.	12s	-	+	+	+	+	+	+	+	-	-	-	-	-	-	-	-
68.	13s	-	+	+	+	+	+	+	+	+	+	+	+	+	-	-	-
69.	14s	-	+	+	+	+	+	+	-	-	-	-	-	-	-	-	-
70.	18s	-	+	+	+	+	+	+	+	+	+	+	+	+	+	+	+
71.	19s	-	+	+	+	+	+	+	-	-	-	-	-	-	-	-	-
72.	19s	-	+	+	+	+	+	+	+	+	+	+	+	+	+	+	+
73.	1s	-	+	+	+	+	+	+	-	-	-	-	-	-	-	-	-
74.	20s	-	+	+	+	+	+	+	+	-	-	-	-	-	-	-	-
75.	21s	-	+	+	+	+	+	+	+	-	-	-	-	-	-	-	-
76.	22s	-	+	+	+	+	+	+	+	-	-	-	-	-	-	-	-
77.	23s	-	+	+	+	+	+	+	+	+	+	+	+	+	-	-	-
78.	25s	-	+	+	+	+	+	+	-	-	-	-	-	-	-	-	-
79.	26s	-	+	+	+	+	+	+	+	+	+	+	+	-	-	-	-
80.	2s	-	+	+	+	+	+	+	+	-	-	-	-	-	-	-	-
81.	31s	-	+	+	+	+	+	+	+	+	+	+	-	-	-	-	-
82.	34s	-	+	+	+	+	+	+	+	+	-	-	-	-	-	-	-
83.	39s	-	+	+	+	+	+	+	+	+	-	-	-	-	-	-	-
84.	3s	-	+	+	+	+	+	+	+	+	-	-	-	-	-	-	-
85.	40s	-	+	+	+	+	+	+	+	+	+	+	+	+	+	-	-
86.	41s	-	+	+	+	+	+	+	+	+	+	+	+	+	-	-	-
87.	42s	-	+	+	+	+	+	+	+	+	+	+	-	-	-	-	-
88.	43s	-	+	+	+	+	+	+	+	+	+	-	-	-	-	-	-
89.	44s	-	+	+	+	+	+	+	+	-	-	-	-	-	-	-	-
90.	45s	-	+	+	+	+	+	+	+	+	+	+	+	+	+	+	+
91.	4s	-	+	+	+	+	+	+	-	-	-	-	-	-	-	-	-
92.	50s	-	+	+	+	+	+	+	+	+	+	+	+	+	+	+	+
93.	52s	-	+	+	+	+	+	+	+	+	-	-	-	-	-	-	-
94.	56s	-	+	+	+	+	+	+	+	+	+	+	+	-	-	-	-
95.	57s	-	+	+	+	+	+	+	-	-	-	-	-	-	-	-	-
96.	59s	-	+	+	+	+	+	+	+	+	-	-	-	-	-	-	-
97.	60s	-	+	+	+	+	+	+	+	+	-	-	-	-	-	-	-
98.	65s	-	+	+	+	+	+	+	+	+	+	+	+	+	+	+	+
99.	67s	-	+	+	+	+	+	+	-	-	-	-	-	-	-	-	-

100.	68s	-	+	+	+	+	+	+	+	+	-	-	-	-	-	-	-
101.	6s	-	+	+	+	+	+	+	+	+	-	-	-	-	-	-	-
102.	82s	-	+	+	+	+	+	+	-	-	-	-	-	-	-	-	-
103.	86s	-	+	+	+	+	+	+	+	+	+	+	-	-	-	-	-
104.	87s	-	+	+	+	+	+	+	+	+	+	-	-	-	-	-	-
105.	88s	-	+	+	+	+	+	+	-	-	-	-	-	-	-	-	-
106.	97s	-	+	+	+	+	+	+	+	+	+	+	+	+	+	+	+
107.	9s	-	+	+	+	+	+	+	+	+	+	+	+	+	+	+	+
108.	A3	-	+	-	-	-	-	-	-	-	-	-	-	-	-	-	-
109.	A4	-	+	-	-	-	-	-	-	-	-	-	-	-	-	-	-
110.	2t	-	+	+	+	+	+	+	+	+	+	+	+	+	-	-	-
111.	3t	-	+	+	+	+	+	+	+	+	+	+	+	+	+	+	-
112.	5t	-	+	+	+	+	+	+	+	+	+	+	+	+	-	-	-
113.	6t	-	+	+	+	+	+	+	+	+	+	-	-	-	-	-	-
114.	7t	-	+	+	+	+	+	+	+	+	+	-	-	-	-	-	-
115.	11t	-	+	+	+	+	+	+	+	+	+	+	+	+	-	-	-
116.	12t	-	+	+	+	+	+	+	+	+	-	-	-	-	-	-	-
117.	13t	-	+	+	+	+	+	+	+	+	+	+	+	+	+	+	-
118.	14t	-	+	+	+	+	+	+	+	+	-	-	-	-	-	-	-
119.	15t	-	+	+	+	+	+	+	+	+	+	+	+	+	+	-	-
120.	16t	-	+	+	+	+	+	+	+	+	-	-	-	-	-	-	-
121.	17t	-	+	+	+	+	+	+	+	+	-	-	-	-	-	-	-
122.	18t	-	+	+	+	+	+	+	+	+	+	+	+	+	+	+	-
123.	19t	-	+	+	+	+	+	+	+	-	-	-	-	-	-	-	-
124.	21t	-	+	+	+	+	+	+	+	-	-	-	-	-	-	-	-
125.	22t	-	+	+	+	+	+	+	+	-	-	-	-	-	-	-	-
126.	23t	-	+	+	+	+	+	+	-	-	-	-	-	-	-	-	-
127.	24t	-	+	+	+	+	+	+	+	+	+	+	+	+	+	+	+
128.	25t	-	+	+	+	+	+	+	+	+	+	-	-	-	-	-	-
129.	26t	-	+	+	+	+	+	+	+	+	-	-	-	-	-	-	-
130.	27t	-	+	+	+	+	+	+	+	+	+	+	+	+	+	-	-
131.	28t	-	+	+	+	+	+	+	+	+	+	-	-	-	-	-	-
132.	29t	-	+	+	+	+	+	+	-	-	-	-	-	-	-	-	-
133.	34t	-	+	+	+	+	+	+	-	-	-	-	-	-	-	-	-

134.	35t	-	+	+	+	+	+	+	+	+	-	-	-	-	-	-	-
135.	36t	-	+	+	+	+	+	+	+	-	-	-	-	-	-	-	-
136.	37t	-	+	+	+	+	+	+	+	-	-	-	-	-	-	-	-
137.	38t	-	+	+	+	+	+	+	+	+	+	-	-	-	-	-	-
138.	39t	-	+	+	+	+	+	+	+	-	-	-	-	-	-	-	-
139.	40t	-	+	+	+	+	+	+	+	+	+	+	+	+	+	-	-
140.	41t	-	+	+	+	+	+	+	+	+	+	-	-	-	-	-	-
141.	43t	-	+	+	+	+	+	+	+	+	+	-	-	-	-	-	-
142.	44t	-	+	+	+	+	+	+	+	+	+	+	+	+	-	-	-
143.	48t	-	+	+	+	+	+	+	-	-	-	-	-	-	-	-	-
144.	49t	-	+	+	+	+	+	+	-	-	-	-	-	-	-	-	-
145.	50t	-	+	+	+	+	+	+	+	+	+	-	-	-	-	-	-
146.	51t	-	+	+	+	+	+	+	+	-	-	-	-	-	-	-	-
147.	52t	-	+	+	+	+	+	+	+	+	-	-	-	-	-	-	-
148.	53t	-	+	+	+	+	+	+	+	-	-	-	-	-	-	-	-
149.	54t	-	+	+	+	+	+	+	+	+	-	-	-	-	-	-	-
150.	55t	-	+	+	+	+	+	+	+	+	+	+	+	-	-	-	-
151.	56t	-	+	+	+	+	+	+	+	+	+	+	+	+	-	-	-
152.	59t	-	+	+	+	+	+	+	+	-	-	-	-	-	-	-	-
153.	60t	-	+	+	+	+	+	+	+	-	-	-	-	-	-	-	-
154.	61t	-	+	+	+	+	+	+	-	-	-	-	-	-	-	-	-
155.	67t	-	+	+	+	+	+	+	+	+	-	-	-	-	-	-	-
156.	69t	-	+	+	+	+	+	+	+	+	+	+	+	-	-	-	-
157.	70t	-	+	+	+	+	+	+	+	+	+	+	+	-	-	-	-
158.	71t	-	+	+	+	+	+	+	-	-	-	-	-	-	-	-	-
159.	74t	-	+	+	+	+	+	+	+	+	+	+	+	+	+	+	+
160.	75t	-	+	+	+	+	+	+	-	-	-	-	-	-	-	-	-
161.	77t	-	+	+	+	+	+	+	+	+	+	+	+	+	+	-	-
162.	78t	-	+	+	+	+	+	+	+	+	+	+	+	+	+	+	+
163.	80t	-	+	+	+	+	+	+	-	-	-	-	-	-	-	-	-
164.	86t	-	+	+	+	+	+	+	-	-	-	-	-	-	-	-	-
165.	87t	-	+	+	+	+	+	+	+	-	-	-	-	-	-	-	-
166.	88t	-	+	+	+	+	+	+	+	+	+	+	+	+	+	+	+
167.	89t	-	+	+	+	+	+	+	+	+	+	+	+	+	+	+	-

168.	92t	-	+	+	+	+	+	+	+	+	-	-	-	-	-	-	-
169.	95t	-	+	+	+	+	+	+	+	+	-	-	-	-	-	-	-
170.	97t	-	+	+	+	+	+	+	+	+	-	-	-	-	-	-	-
171.	99t	-	+	+	+	+	+	+	+	-	-	-	-	-	-	-	-
172.	10st	-	+	+	+	+	+	+	+	-	-	-	-	-	-	-	-
173.	11st	-	+	+	+	+	+	+	+	+	-	-	-	-	-	-	-
174.	12st	-	+	+	+	+	+	+	+	-	-	-	-	-	-	-	-
175.	13st	-	+	+	+	+	+	+	+	+	+	+	+	+	-	-	-
176.	14st	-	+	+	+	+	+	+	-	-	-	-	-	-	-	-	-
177.	18st	-	+	+	+	+	+	+	+	+	+	+	+	+	+	+	+
178.	19st	-	+	+	+	+	+	+	-	-	-	-	-	-	-	-	-
179.	19st	-	+	+	+	+	+	+	+	+	+	+	+	+	+	+	+
180.	1st	-	+	+	+	+	+	+	-	-	-	-	-	-	-	-	-
181.	20st	-	+	+	+	+	+	+	+	-	-	-	-	-	-	-	-
182.	21st	-	+	+	+	+	+	+	+	-	-	-	-	-	-	-	-
183.	22st	-	+	+	+	+	+	+	+	-	-	-	-	-	-	-	-
184.	23st	-	+	+	+	+	+	+	+	+	+	+	+	+	-	-	-
185.	25st	-	+	+	+	+	+	+	-	-	-	-	-	-	-	-	-
186.	26st	-	+	+	+	+	+	+	+	+	+	+	+	-	-	-	-
187.	2st	-	+	+	+	+	+	+	+	-	-	-	-	-	-	-	-
188.	31st	-	+	+	+	+	+	+	+	+	+	+	-	-	-	-	-
189.	34st	-	+	+	+	+	+	+	+	+	-	-	-	-	-	-	-
190.	39st	-	+	+	+	+	+	+	+	+	-	-	-	-	-	-	-
191.	3st	-	+	+	+	+	+	+	+	+	-	-	-	-	-	-	-
192.	40st	-	+	+	+	+	+	+	+	+	+	+	+	+	+	-	-
193.	41st	-	+	+	+	+	+	+	+	+	+	+	+	+	-	-	-
194.	42st	-	+	+	+	+	+	+	+	+	+	+	-	-	-	-	-
195.	43st	-	+	+	+	+	+	+	+	+	+	-	-	-	-	-	-
196.	44st	-	+	+	+	+	+	+	+	-	-	-	-	-	-	-	-
197.	45st	-	+	+	+	+	+	+	+	+	+	+	+	+	+	+	+
198.	4st	-	+	+	+	+	+	+	-	-	-	-	-	-	-	-	-
199.	50st	-	+	+	+	+	+	+	+	+	+	+	+	+	+	+	+
200.	52st	-	+	+	+	+	+	+	+	+	-	-	-	-	-	-	-
201.	56st	-	+	+	+	+	+	+	+	+	+	+	+	-	-	-	-

**202.**	**57st**	-	+	+	+	+	+	+	-	-	-	-	-	-	-	-	-
**203.**	**59st**	-	+	+	+	+	+	+	+	+	-	-	-	-	-	-	-
**204.**	**60st**	-	+	+	+	+	+	+	+	+	-	-	-	-	-	-	-
**205.**	**65st**	-	+	+	+	+	+	+	+	+	+	+	+	+	+	+	+
**206.**	**67st**	-	+	+	+	+	+	+	-	-	-	-	-	-	-	-	-
**207.**	**68st**	-	+	+	+	+	+	+	+	+	-	-	-	-	-	-	-
**208.**	**6st**	-	+	+	+	+	+	+	+	+	-	-	-	-	-	-	-
**209.**	**82st**	-	+	+	+	+	+	+	-	-	-	-	-	-	-	-	-
**210.**	**86st**	-	+	+	+	+	+	+	+	+	+	+	-	-	-	-	-
**211.**	**87st**	-	+	+	+	+	+	+	+	+	+	-	-	-	-	-	-
**212.**	**88st**	-	+	+	+	+	+	+	-	-	-	-	-	-	-	-	-
**213.**	**97st**	-	+	+	+	+	+	+	+	+	+	+	+	+	+	+	+
**214.**	**9st**	-	+	+	+	+	+	+	+	+	+	+	+	+	+	+	+

Keys:

C1 = control I

NEGATIVE CONTROL (serum + PBS)

C2 = control II   POSITIVE CONTROL (serum + vaccine)

A1=NORMAL PERSON 1

A2=NORMAL PERSON 2

+ = water droplet formation

- = no water droplet formation

Supplementary Table S2: Different type of negative and positive used to develop thin layer immunoassay.

S. No	Control samples	
	**Negative controls**	**Positive controls**
1.	*H. pylori* antigens = 100ul of 0.02025mg/ml	Human gastric patients serum = 100ul of0.36mg/ml
2.	Phosphate Buffer saline (washing solution) = 100ul	
3.	Secondary antibody = 100ul	*H. pylori* immunized rabbit serum = 100ul of0.36mg/ml
4.	Control normal rabbit serum = 100ul of 0.24mg/ml	

5.	Control normal sheep Serum = 100ul of 0.32mg/ml	*H. pylori* immunized mice serum = 100ul of0.36mg/ml
6.	Human serum of normal person = 100ul of 0.1mg/ml	

Supplementary Table S3: Anti-*H. pylori* IgM antibody titer values in gastroduodenal ulcer and gastritis patients by IgM commercial kit ELISA (k_MELISA)[30].

Negative controls (Optical Density)	0.015	Positive controls (Optical Density)	1.405
	0.014		1.157
	0.0106		1.074
Mean ± SD	0.013 ± 0.002	Mean ± SD	1.212 ± 0.172
Cutoff value = Mean of negative control + 0.250			0.263
Prevalence of *H. pylori* by IgM ELISA kit			75 % (0.921 ± 0.633)

Supplementary Table S4: Anti- *H. pylori* IgG antibody titer values in gastroduodenal ulcer and gastritis patients by IgG commercial kit ELISA (k_GELISA)[30]

Negative controls (Optical Density)	0.0037	Positive controls (Optical Density)	1.006
	0.0032		1.179
	0.0038		1.083
Mean ± SD	0.003 ± 0.0003	Mean ± SD	1.089 ± 0.0866
Cutoff value = Mean of negative control + 0.250			0.253
Prevalence of *H. pylori* by IgG ELISA kit			94 % (2.144 ± 0.997)
